# Sex differences in the representation of call stimuli in a songbird secondary auditory area

**DOI:** 10.3389/fnbeh.2015.00290

**Published:** 2015-10-28

**Authors:** Nicolas Giret, Fabien Menardy, Catherine Del Negro

**Affiliations:** Department Cognition and Behaviors, Paris-Saclay Institute of Neuroscience, Centre National de la Recherche Scientifique UMR 9197, Paris-Sud UniversityOrsay, France

**Keywords:** auditory perception, sexual dimorphism, zebra finch, response properties, single-unit recording, neural code, vocal communication, discrimination

## Abstract

Understanding how communication sounds are encoded in the central auditory system is critical to deciphering the neural bases of acoustic communication. Songbirds use learned or unlearned vocalizations in a variety of social interactions. They have telencephalic auditory areas specialized for processing natural sounds and considered as playing a critical role in the discrimination of behaviorally relevant vocal sounds. The zebra finch, a highly social songbird species, forms lifelong pair bonds. Only male zebra finches sing. However, both sexes produce the distance call when placed in visual isolation. This call is sexually dimorphic, is learned only in males and provides support for individual recognition in both sexes. Here, we assessed whether auditory processing of distance calls differs between paired males and females by recording spiking activity in a secondary auditory area, the caudolateral mesopallium (CLM), while presenting the distance calls of a variety of individuals, including the bird itself, the mate, familiar and unfamiliar males and females. In males, the CLM is potentially involved in auditory feedback processing important for vocal learning. Based on both the analyses of spike rates and temporal aspects of discharges, our results clearly indicate that call-evoked responses of CLM neurons are sexually dimorphic, being stronger, lasting longer, and conveying more information about calls in males than in females. In addition, how auditory responses vary among call types differ between sexes. In females, response strength differs between familiar male and female calls. In males, temporal features of responses reveal a sensitivity to the bird's own call. These findings provide evidence that sexual dimorphism occurs in higher-order processing areas within the auditory system. They suggest a sexual dimorphism in the function of the CLM, contributing to transmit information about the self-generated calls in males and to storage of information about the bird's auditory experience in females.

## Introduction

The social behavior of many species suggests a capacity for the recognition of individuals. Variations in vocalizations provide a basis for this ability in many vocally communicating species. To date, identifying the neural basis of the capacity to distinguish vocalizations that differ among individuals remains a challenge. Songbirds provide an attractive animal model to address this issue (Gentner, 2004). They produce a set of acoustically complex vocalizations, songs, and calls, that may convey information regarding identity through both species- and individual-specific features (Falls, 1982; Lambrechts and Dhondt, 1995). As in humans, the production, and discrimination of their vocal communication signals depend on learned processes, and could be adapted to auditory and social contexts (Doupe and Kuhl, 1999).

As animal models in which to study the general principles of the neural coding of communication sounds (Theunissen and Elie, 2014), songbirds provide an opportunity for comparative studies (Brenowitz, 1997). They constitute one of the best-known examples of sexual dimorphism in the vertebrate brain. In particular, in species in which males and females differ in their vocal behavior (but not exclusively, see Gahr, 2007; Gahr et al., 2008), the nuclei involved in song production and learning are larger in males than in females and may exhibit sex differences in neuron number, cell size, or dendritic length (Ball and MacDougall-Shackleton, 2001; see review in MacDougall-Shackleton and Ball, 1999). Moreover, the physiological and functional properties of neurons, in particular the encoding of conspecific songs, have been reported to differ between males and females (Del Negro et al., 2000; Del Negro and Edeline, 2001; Liu et al., 2010). Functional differences studied by lesioning suggest that song nuclei, in particular the premotor vocal area, nucleus HVC (used as a proper noun) may serve, to a certain extent, distinct functions in male and female songbirds (Brenowitz, 1991; Del Negro et al., 1998).

In contrast to song nuclei, the ascending circuits that process auditory signals do not show any pronounced anatomical sexual dimorphism. This ascending pathway conveys auditory information from the cochlea to telencephalic areas that includes Field L, the first post-thalamic processing stage, and its direct or indirect targets, the caudal nidopallium (NCM) and the caudal mesopallium (CM), that in turn can be subdivided into the caudomedial and caudolateral mesopallium (CMM and CLM, respectively) (Vates et al., 1996; Bolhuis and Gahr, 2006). Pallial auditory areas contribute not only to the auditory perception of natural communication sounds, but also play a role in memory formation, which is required to continuously adapt to the social environment (Bolhuis and Gahr, 2006; Pinaud and Terleph, 2008; Hahnloser and Kotowicz, 2010). Neurons in the NCM and CM exhibit learning-dependent responses and may be particularly sensitive to behaviorally relevant vocalizations, such as those produced by familiar individuals (Gentner and Margoliash, 2003; Thompson and Gentner, 2010; Jeanne et al., 2011).

In spite of the lack of marked anatomical sexual dimorphism, central auditory areas may show sex differences in the neuronal activation induced by vocalizations, including calls and songs. In black-capped chickadees (*Poecile atricapillus*), exposure to song or call induces an increase in immediate early gene (ZENK) expression in both the NCM and CMM that is greater in males than in females (Phillmore et al., 2003; Avey et al., 2008), although males and females both produce the two types of vocalizations. In zebra finches (*Taeniopygia guttata*), changes in gene expression in the NCM and CMM in response to female call playback are only observed in females, although male and females behaviorally respond to call playback with a clear preference for those produced by females (Gobes et al., 2009). These striking differences could result from sexual dimorphism at the cellular level (Pinaud et al., 2006), and suggest that sex differences do occur in the encoding of vocal communication sounds by auditory brain circuits. However, this hypothesis remains largely to be explored.

In the present study, we investigated the possibility of sex differences in the representation of vocal communication sounds in one pallial auditory region, the CLM, of zebra finches. This region contains neurons that exhibit selectivity for conspecific songs compared to synthetic sounds (Sen et al., 2001; Hsu et al., 2004; Jeanne et al., 2011; Meliza and Margoliash, 2012) and that respond to call playback (Elie and Theunissen, 2015). In male zebra finches, the CLM is considered as being well suited to convey auditory feedback information crucial to song learning and maintenance: neurons are active not only in response to song playback but also during singing and some of them are sensitive to feedback perturbations (Bauer et al., 2008; Keller and Hahnloser, 2009). Since female zebra finches do not sing and are only faced with the tasks of song perception and discrimination, neuronal processing in the CLM could differ between males and females.

We therefore examined the response properties of CLM neurons in male and female zebra finches using a variety of distance calls. In this highly social species, this call type is frequently produced by both sexes, especially when they lose visual contact with their social partners (Zann, 1996; Vicario et al., 2001, 2002). In males, the distance call requires a learning process (Simpson and Vicario, 1990; Gentner and Margoliash, 2003; Forstmeier et al., 2009; Jeanne et al., 2011). Also, it is frequently incorporated as a syllable in the song. Therefore, given the potential role of CLM in the transmission of information about singing-related auditory feedback (Bauer et al., 2008; Keller and Hahnloser, 2009), neural processing of distance calls could differ between males and females. The distance call also encodes individual identity and supports the recognition of the mate and familiar individuals (Vignal et al., 2004, 2008; Vignal and Mathevon, 2011; Perez et al., 2012). A recent study has reported that, in starlings (*Sturnus vulgaris*), learning may affect the neuronal encoding of song stimuli in the CLM, increasing the amount of information provided by individual neurons in their responses to song playbacks (Jeanne et al., 2011). This led us to expose CLM neurons to male and female calls that differed in their degree of familiarity. Individuals had experienced the call of their mate for several months and the calls of familiar individuals for several days. The sets of stimuli used included the bird's own call to examine whether the neuronal processing of this self-generated vocalization differs between males and females.

## Methods

### Subjects and housing conditions

The subjects were adult zebra finches (*T. guttata*), seven males and seven females, reared socially in the breeding colony of the Paris-Sud University. Birds were kept under a 12:12 light-dark cycle, with food and water *ad libitum*, and an ambient temperature of 22–25°C. About 4 months prior to the experiment, seven pairs were formed and placed in individual cages (dimensions 24 × 29 × 39 cm). All pairs had raised at least one clutch of offspring by the time of the electrophysiological investigation. To determine whether the degree of familiarity affects call-evoked responses in CLM neurons, we familiarized mated individuals with the distance calls of the partners of two other pairs. To this end, each pair of experimental birds was placed in a new cage allowing close visual and auditory interactions with two other pairs at least 5 days prior to the electrophysiological investigation. Because experimental birds were kept in presence of neighbors until the electrophysiological experiment started, none of the experimental pairs were used as “familiar” pairs and inversely. Adult zebra finches are able to discriminate among songs of different individuals (Miller, 1979a,b; Clayton, 1988) and to form memories of specific songs after hearing them for only 3 h in non-reinforced playback (Stripling et al., 2003). Even though our study focused on another type of vocalization, we assumed that these behavioral conditions would allow individuals to become familiar with the call of neighbors.

Experimental procedures were carried out in compliance with national (JO 887–848) and European (86/609/EEC) legislation on animal experimentation, and following the guidelines used by the animal facilities of Paris-Sud University (Orsay, France), approved by the national directorate of veterinary services (# D91-429).

### Auditory stimuli

The sets of distance calls used as auditory stimuli included the calls produced by the two sexual partners of three categories of bird pairs: the experimental pair, i.e., the bird in which the activity of CLM neurons was recorded and its sexual partner, the two neighboring pairs (“familiar calls”; FAM1 and FAM2; see Table 1; 14 female calls from 14 different females and 14 male calls from 14 different males) and two unfamiliar pairs (“unfamiliar calls”; UNFAM1 and UNFAM2; total number of calls used as stimuli: 14 female calls from 14 different females and 14 male calls from 14 different males). Hence, sets included six types of call stimuli and a total of 10 distinct calls (given that each type except both the mate's call and the bird's own call was represented by two distinct calls; see Table 1). The UNFAM call stimuli were drawn from a collective pool of 14 UNFAM recordings, and all experimental pairs were tested with a different subset of this pool. Some of them were used as call stimuli in previous experiments (Menardy et al., 2012, 2014). Two factors therefore distinguished the calls used as auditory stimuli: the sex of the vocalizer and the category (mate/bird's own call, familiar and unfamiliar; see Table 1). The same set of call stimuli was presented to the two partners of each experimental pair, with the mate's call of one partner being the bird's own call of the other partner.

**Table 1:** **Mean duration of calls used as acoustic stimuli**.

**Acoustic stimuli**		**Types**	**Duration (ms; mean ± SD)**
Calls	F-BOC/F-Mate	Call of the female (Bird's own call/Mate's call)	237 ± 44.0
	F-FAM (two calls per pair; F-FAM 1 and F-FAM2)	Calls of familiar females	310 ± 28.3
	F-UNFAM (two calls per pair; F-UNFAM 1 and F-UNFAM2)	Calls of unfamiliar females	300 ± 10.0
	M-BOC/M-Mate	Call of the male (Bird's own call/Mate's call)	177 ± 38.7
	M-FAM (two calls per pair; M-FAM 1 and M-FAM2)	Calls of familiar males	230 ± 70.7
	M-UNFAM (two calls per pair; M-UNFAM 1 and M-UNFAM2)	Calls of unfamiliar males	250 ± 42.4

To record distance calls, the individual was separated from its partner and housed individually in a small cage placed in a sound-attenuating chamber. All birds (experimental, familiar, or unfamiliar) were recorded in isolation under the same conditions. Calls were recorded using a Sennheiser MD 46 microphone (Sennheiser Electronic, Wedemark, Germany) connected to a Marantz PMD670 recorder with a 44 kHz sampling rate, and were analyzed off-line using Avisoft software (Avisoft SASLabPro, Berlin, Germany). From the recorded calls of a given bird, we paid attention to select one representative exemplar on the basis of a visual inspection of call structure. The female distance call consists of a harmonic series with a fundamental frequency of 400–500 Hz (Simpson and Vicario, 1990; Vicario et al., 2002; Vignal and Mathevon, 2011). It can be divided into three segments of different durations: the initial segment defined by a short and loud ascending frequency modulation, the stable segment defined by a long and loud plateau with no frequency modulation, and a third segment defined by a short and weak descending frequency modulation (Vignal et al., 2004). The duration of female calls used as sound stimuli varied from 190 to 390 ms, with the mean duration of calls in each category used as auditory stimuli indicated in Table 1. Male distance calls can be distinguished from female calls by, at least, three features: a higher fundamental frequency of 650–1000 Hz (Vicario et al., 2001; Vignal et al., 2008; Vignal and Mathevon, 2011), a segment with a rapid frequency modulation and a shorter duration (Vignal and Mathevon, 2011). However, as previously reported (Simpson and Vicario, 1990), the distance call of certain males lacks one or more male-typical features, in particular the fast frequency modulated segment (see examples of male calls in Simpson and Vicario, 1990; Menardy et al., 2012). The duration of male distance calls used as auditory stimuli varied from 150 to 320 ms. In addition, the acoustic structure of the distance call varies according to the identity of the caller and supports discrimination among conspecifics and recognition by the mate (Zann, 1984; Vignal et al., 2004; Forstmeier et al., 2009; Vignal and Mathevon, 2011; Perez et al., 2012).

### Electrophysiological recording

Birds were anesthetized with isoflurane gas (in oxygen; induction: 3%, maintenance: 1.5%) that flowed through a small mask over the bird's beak. In a sound attenuation chamber (chamber model AC2; IAC, New York, NY), the bird was immobilized in a custom-made stereotaxic holder that allowed the head to be tilted at 45°. Lidocaine cream was applied to the skin. A window was opened in the inner skull layer and small incisions were made in the dura. A high impedance tungsten microelectrode (10–12 MΩ; FHC, Inc. Bowdoin, ME, USA) was lowered into the brain. The coordinates used were in most cases 1.3 mm anterior (range: 1.2–1.5 mm) and 1.2 mm lateral (range: 1.0–1.4 mm) to the bifurcation of the sagittal sinus and 1.2 mm deep (range: 0.7–1.9 mm). These coordinates, close to those used by Bauer et al. (2008), correspond to the medial part of the caudolateral hyperstriatum ventrale in Vates et al. (1996). When the neural trace was dominated by one individual neuron, sound stimuli were delivered. Recording sites were at least 100 μm apart to guarantee that the neural activity recorded from two successive sites originated from different single units. The neural signal was amplified (gain 5000; bandpass: 0.3–10 kHz), monitored on-line by oscilloscope and sent in parallel to an audio monitor. The signal was digitized by a data acquisition system (CED Power 1401 interface; Cambridge, UK) and stored on a personal computer. In parallel, call stimuli were concomitantly recorded using a microphone and digitized by the CED system. This enabled us to precisely determine the onset of the auditory response with respect to the sound stimulus.

While spiking activity was recorded, the whole stimulus set, including male and female calls of the three categories of pairs (the experimental pair, the familiar/neighboring pairs, and unfamiliar pairs) was broadcasted through a speaker situated 30 cm from the bird. From one recording site to the following one, the delivery order of the 10 calls used as auditory stimuli (given that each type of call except both the mate's call and the bird's own call was represented by two distinct calls) varied. Each call stimulus was presented repeatedly to examine whether CLM neurons underwent a modulation in the magnitude of their responses with call repetition that differed between males and females. Both preparation of auditory stimuli, i.e., the selection of only one representative call per individual, and playback procedure were similar as those used in previous experiments (Menardy et al., 2012, 2014). Each call stimulus was presented successively 50 times. These 50 presentations consisted of 5 blocks of 10 repetitions with an inter-stimulus interval of 1 s within a block and of 5 s between two blocks. An inter-iteration interval of 1 s is within the range of spontaneous call recurrence during vocal interaction in zebra finches (Beckers and Gahr, 2010). A silence of 30 s separated the playback of two different call stimuli. All stimuli were normalized to achieve a maximal amplitude of 65–70 dB (Avisoft software) at the level of the bird.

### Data processing and analysis

The overall quality of neuronal recordings, as indicated by spike amplitude relative to the background noise, was the first criterion used to analyze spiking activity. Subsequently, only neural traces that were dominated by the activity of one or two neurons were subjected to template-based spike detection and sorting (Spike2 software, version 7, CED, Cambridge, UK). Spike event times were binned at 10-ms intervals for analysis. For all repetitions of a given call stimulus at a single recording site, a peristimulus histogram (PSTH, 10 ms per bin) was built and then PSTHs were averaged over the 50 trials. Spiking activity was first analyzed by calculating spontaneous activity (defined as the mean frequency of spikes generated in the last 500 ms preceding call presentation). We also measured spike frequency during and for 50 ms following call stimulus presentation.

To estimate the strength of the response evoked by a given call stimulus and to further compare responses between males and females, it was important to control for differences in level of spontaneous activity between units and to limit the influence of one or a few very active units. To this end, we normalized each unit's stimulus response (Stripling et al., 1997; Menardy et al., 2012). The response strength (*Ri*) index was calculated by subtracting the spontaneous activity rate (*B*_*FR*;_ calculated over the number of 10 ms bins of the baseline period of the averaged PSTH) from the activity rate generated during stimulus presentation (*S*_*FR*_), and then dividing this value by their sum:

$$\begin{array}{l} R_{i}=\frac{\left(S_{FR}-B_{FR}\right)}{\left(S_{FR}+B_{FR}\right)} \end{array}$$

*Ri* values fall between +1 and -1, where values >0 indicate an excitatory response and values < 0 indicate an inhibitory response.

A nonparametric measure has been used in recent studies to assess response properties of neurons in various telencephalic auditory areas, including the CLM (Jeanne et al., 2011; Meliza and Margoliash, 2012). This nonparametric measure, called activity fraction (AF) or sparseness (Vinje and Gallant, 2000; Lehky et al., 2005), is an index calculated from the response of a given neuron to each of the *n* stimuli. We used the formula:

$$\begin{array}{l} AF=\left[1-{\left[\overset{n}{\underset{i=1}{\sum }}f_{i}\;∕n\right]}^{2}∕\overset{n}{\underset{i=1}{\sum }}\left(f_{i}^{2}\;∕n\right)\right]∕\left(1-1∕\text{n}\right) \end{array}$$

where f_*i*_ is the mean firing rate of the neuron in response to the *i*^*th*^ stimulus, averaged across iterations, and *n* is the total number of call stimuli. The index is 0 if the neuron shows equivalent responses to all stimuli and 1 if it responds to only one.

To evaluate the degree of excitation driven by a given call stimulus on the basis of a temporal characteristic, we quantified the duration over which spiking activity was significantly increased relative to the call duration. To this end, we calculated the cumulative number of 10 ms bins during which spiking activity exceeded the mean spontaneous firing rate (*B*_*FR*_) by at least 2 standard deviations (SD; calculated over the number of 10 ms bins of the baseline period of the averaged PSTH) and divided this by the total number of bins covered by the call stimulus.

To characterize the profile of the response to a given call stimulus, we assigned responses to two categories: phasic or sustained. In response to the playback of a given call stimulus, some units showed a phasic increase in their activity at stimulus onset that was followed by a rapid decline in firing rate while others exhibited sustained activation over the entire duration of the call stimulus. First, the following criteria were used to identify a change in firing rate as a call-evoked response: an arbitrary *Ri* value >0.15 and at least one 10 ms bin during which spiking activity exceeded the baseline level by 2 SD. Then, we calculated the mean firing rate during the first and the second halves of call presentation and compared these two values with the spontaneous firing rate during a similar period preceding call onset. Responses were considered as being phasic when a statistically significant increase in activity was observed only during the first half of call presentation, and sustained when the neuron still continued to fire above the baseline level during the second half.

To investigate whether the temporal patterns of spike trains differed between males and females, we quantified the amount of mutual information (MI) in neuronal responses, using an indirect method (Schnupp et al., 2006). Briefly, it allows us to quantify how well the identity of the sound stimulus can be inferred from spike trains. As a first step, peristimulus time histograms (PSTH; 10 ms bin width) were created on a neuron-by-neuron and a iteration-by-iteration basis. Because female and male calls differed in their duration, MI values were calculated after separating responses to male calls from those to female calls. The length of the spike trains used for analysis was respectively 200 and 300 ms following call onset in males and females. Each response pattern, i.e., each spike train, was converted into a list of spike count values; these can be thought of as a vector in a multidimensional space and one can quantify how similar two response patterns are by calculating the Euclidean distance between these two responses in this space. Each response in turn was picked as a test pattern and was assigned to the call stimulus that was the closest in terms of Euclidean distance. The accuracy of the classification by the decoder algorithm, i.e., the proportion of assignments to the correct call stimulus, was then calculated. In short, if the response patterns are reproducibly similar among repeated presentations of the same stimulus and reproducibly different from patterns evoked by other stimuli, the response patterns will form distinct clusters in the response space and most patterns will be correctly assigned. However, if the responses lack reproducible and distinctive patterns then the assignment will essentially be random. This procedure was repeated until each trial of a neuron was considered as a test pattern. A confusion matrix allowed an estimation of the MI between neuronal responses and call stimuli. The MI (in bits) is given by Shannon's formula:

$$\begin{array}{l} {MI}_{\left(S;R\right)}=\underset{s,r}{\sum }p\left(s,r\right)\cdot \log_{2}\frac{p\left(s,r\right)}{p\left(s\right)\cdot p\left(r\right)} \end{array}$$

where *s* and *r* are the values obtained by the random variables “presented stimulus class” and “assigned stimulus class.” The *a priori* probability, *p(s)*, of any call stimulus evoking a particular response is 1/5. The probability of a response being assigned to any stimulus class, *p(r)*, and the joint probability of observing a particular combination of stimulus and response assignments, *p(s,r)*, were estimated from the observed frequency distributions in the confusion matrix. We also estimated the expected magnitude of a bias by calculating MI values for “shuffled” data, in which the response patterns had been randomly reassigned to stimulus classes. The shuffling was repeated 20 times and the mean MI estimate for the 20 shuffled datasets was used as an estimator for the bias. Bias estimates varied little from unit to unit, regardless of the call type (male or female calls): the median bias was 0.08 bits per response and it did not exceed 0.17 bits per response. All MI values reported below are “bias-corrected,” i.e., the bias estimate obtained for each unit was subtracted from the original MI estimate. These computations were performed using custom software in the *R* environment.

To quantify the reliability of spike-timing over the renditions of a given stimulus, a measure of correlation between spike trains, the *R*_*corr*_ index, was computed (Schreiber et al., 2003; Huetz et al., 2006). The method involves, first, the convolution of all the spike trains with a Gaussian filter of a given width σ, and then the computation of the inner product between all pairs of iterations, each inner product being divided by the norms of the two iterations of the respective pair. The correlation measure *R*_*corr*_ is the average across all pairs of iterations of the normed inner product:

$$\begin{array}{l} R_{corr}=\frac{2}{n\left(n-1\right)}\overset{n}{\underset{i=1}{\sum }}\overset{n}{\underset{j=i+1}{\sum }}\frac{\vec{S_{i}}\vec{S_{j}}}{∥\vec{S_{i}}∥.∥\vec{S_{j}}∥} \end{array}$$

where *Si* is the convolved spike train, represented as an individual vector and *n* is the stimulus presentation number. The *R*_*corr*_ value, which ranges from 0 (no reliability) to 1 (perfect reliability), quantifies the capacity of a neuron to emit identical spike trains during successive presentations of the same stimulus.

The normality of the variables was assessed with the Liliefors test, revealing a *p* > 0.05 for all dependent variables (a *p* < 0.05 indicating that data are not normally distributed). Responses to call stimuli were appraised by the use of a general linear model (GLM). Firing rate values obtained in response to each stimulus (*n* = 6) for each neuron (*n* = 120) both before (Bfr) and during (Sfr) stimulus presentation were analyzed using a repeated measures (RM) ANOVA in a GLM. We included multiple co-factors in the model: the period (*B*_*FR*_ vs. *S*_*FR*_), the sex of the subject (males vs. females) and the type of the calls (*n* = 6; mate, bird's own call, familiar female, unfamiliar female, familiar male, unfamiliar male) as independent variables and the blocks (*n* = 5) and the trials (*n* = 10) as RM. The *Ri*, response duration and *R*_*corr*_ values were analyzed using RM ANOVA in GLMs that included as co-factors the sex of the subject and the type of call stimulus. For each analysis, as we played back two versions of the familiar and unfamiliar call stimuli to each recorded cells, we averaged the corresponding data. When analyses revealed significant differences, we subjected the data to *post-hoc* Tukey-Kramer tests. The comparison of AF values between males and females was performed using a *t-*test. To determine a sex difference in distribution of neurons within the various categories (phasic, sustained and other), we used a χ^2^-test. The analysis on MI values was performed using a RM ANOVA in a GLM. Also, to examine whether the proportion of correct assignment differed among male call stimuli, we performed a RM ANOVA in a GLM. Statistical computations were carried out in Statistica (Statistica v8.0; StatSoft, Inc.).

### Histology

**Figure 1:**
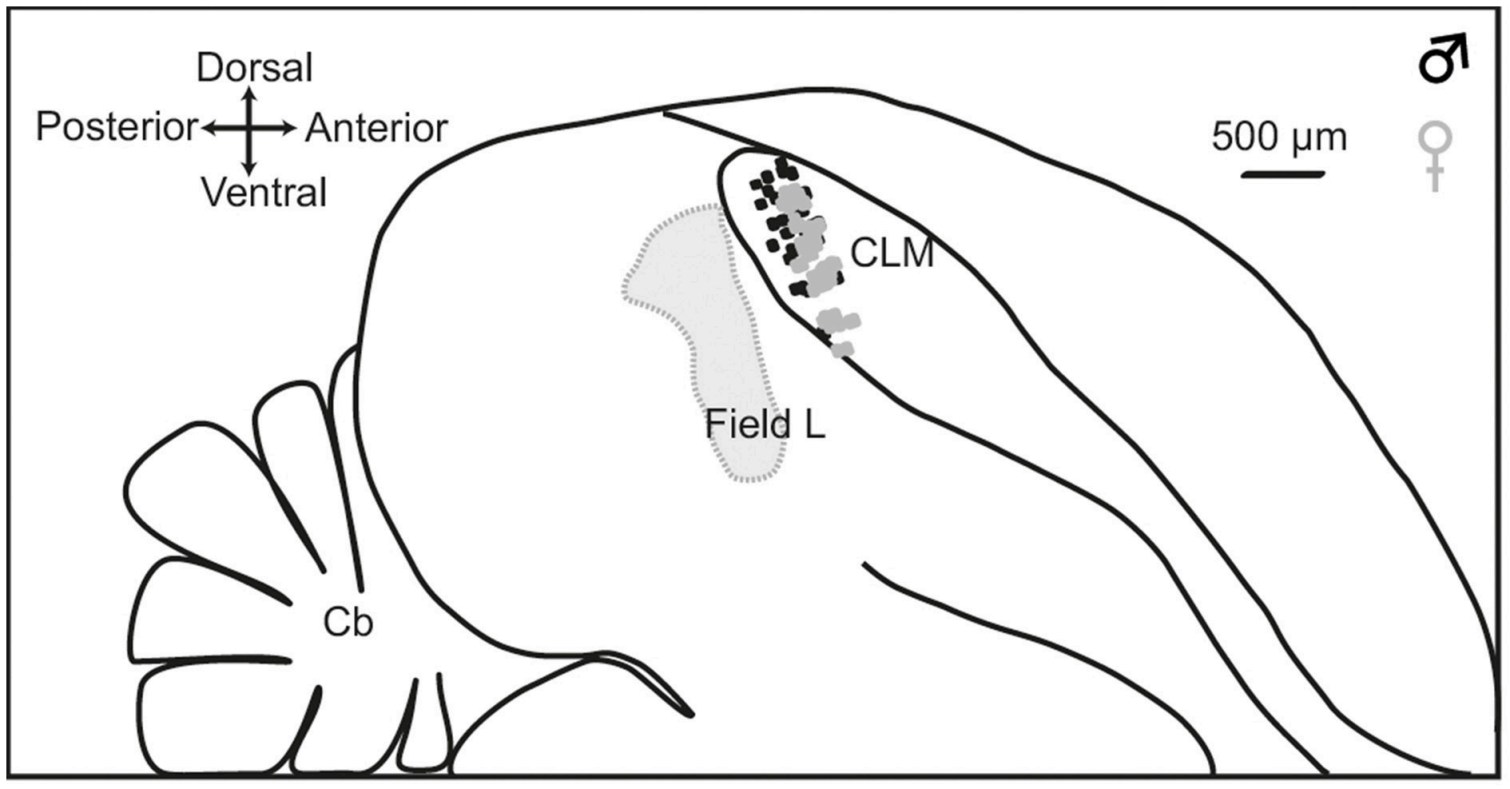
**Location of recording sites**. Outlines are traced from a parasagittal section at 1.2 mm from the midline. Dashed line indicates the boundary of Field L. The CLM region is located ventral to the lateral ventricle and dorsal to Lamina mesopallialis. Dots show the locations of recording sites based on coordinates taken during surgery within this parasagittal plane in males (black dots; number of recording locations, *n* = 56) and females (gray dots; number of recording locations, *n* = 60). CLM, caudal lateral mesopallium; Cb, cerebellum.

At the end of each experiment, the animal was killed with a lethal dose of pentobarbital, the brain quickly removed from the skull and placed in a fixative solution (4% paraformaldehyde) for 2 weeks. Brains were subsequently immersed in 20% sucrose in PBS solution for cryoprotection. Once the brain showed the same density as the sucrose solution (usually after 48 h), sagittal sections (30 μm) were cut on a freezing microtome and processed for Cresyl violet staining. They were examined for electrode penetration tracks. Recording sites were located in the dorsal and medial CLM (Figure 1).

## Results

To examine whether auditory processing in CLM differed between males and females, we recorded extracellular electrophysiological activity in anesthetized males and females of seven pairs of adult zebra finches. Prior to the electrophysiological investigation, males and females had formed a pair bond for at least 4 months. Auditory stimuli included six types of calls: the calls produced by the male and the female of the experimental pair, i.e., the bird's own call and the mate's call, and those produced by males and females of familiar and unfamiliar pairs (see Table 1). Spiking activity was collected from well-isolated single units (in females: *n* = 60, from 6 to 14 per individual; in males: *n* = 60, from 4 to 12 per individual).

### Call-evoked changes in firing rate in males and females

**Figure 2:**
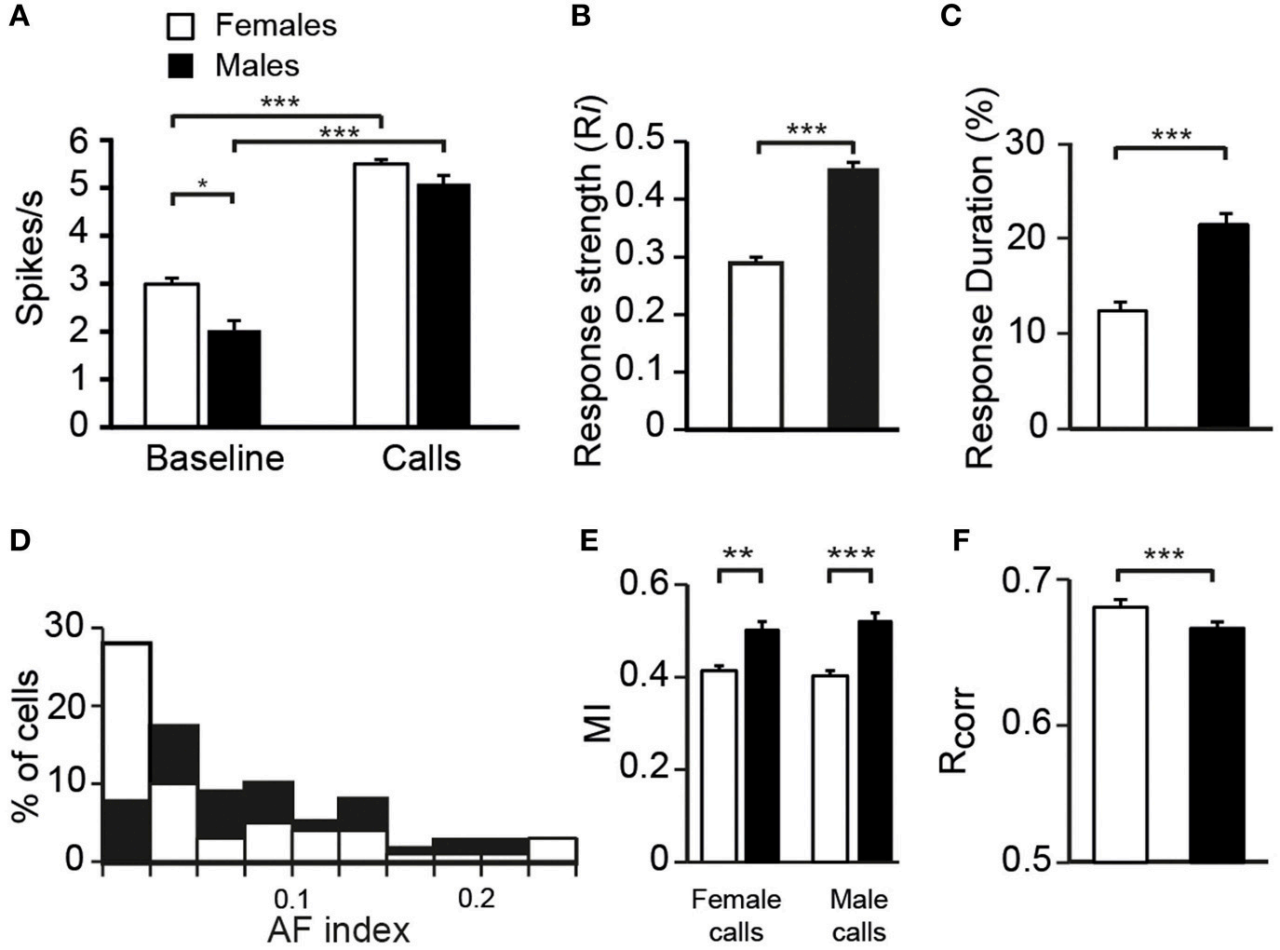
**Differences between females and males in the response properties of neurons**. **(A)** The average spontaneous firing rate (left) is higher in females than in males whereas the average call-evoked firing rate (right) did not differ between sexes. Both the average response strength **(B)** and response duration **(C)** are higher in males than in females. **(D)** The distribution of AF values for neurons recorded in males (black bars) and in females (white bars). The distribution of AF values are significantly different between sexes (χ^2^ = 3.13, *p* < 0.05). **(E)** The amount of mutual information (MI) transmitted in the patterns of responses to female or male calls is higher in males than in females. **(F)** The average values of the correlation index *R*_*corr*_ for females and for males. Each bar represents the mean ± SEM (^*^p < 0.05; ^**^p < 0.01; ^***^p < 0.001).

**Figure 3:**
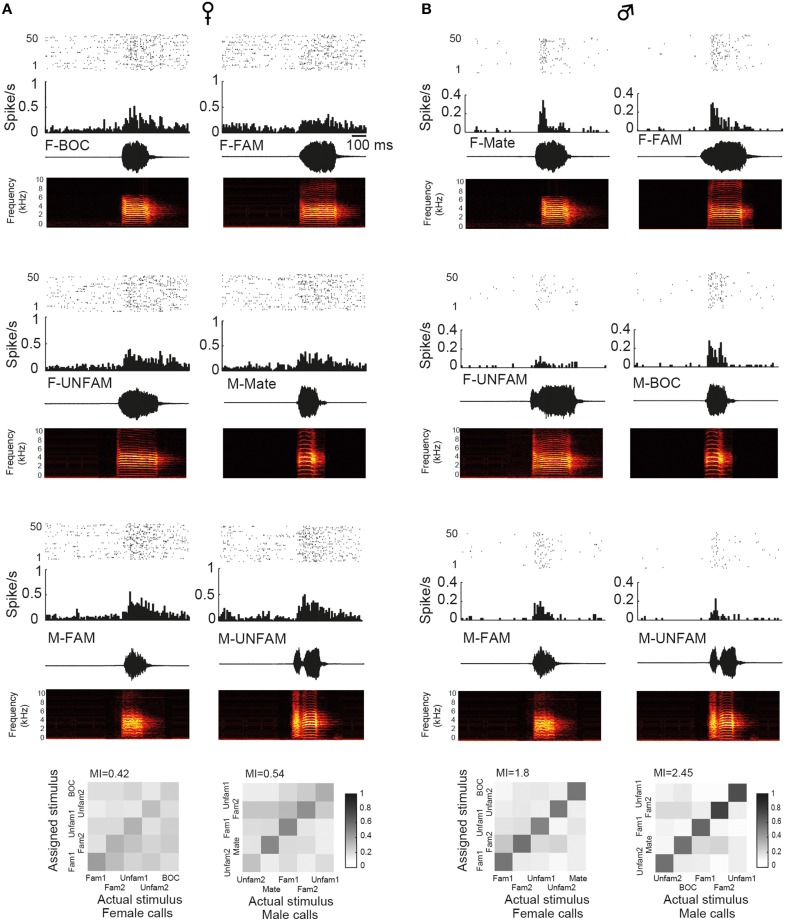
**Examples of single-unit responses obtained in a female (A) and in the male paired with this female (B)**. In each panel are: a raster plot (top 50 iterations), a peristimulus histogram that displays the number of action potentials per bin of 10 ms (middle), and the waveform and spectrogram of the call used as the stimulus (bottom). BOC, bird's own call; F-FAM, call of one familiar female; F-UNFAM, call of one unfamiliar female; MC, mate's call; M-FAM, call of one familiar male; M-UNFAM, call of one unfamiliar male. The calls of two familiar males and females and two unfamiliar males and females were used as auditory stimuli. Neurons have an AF index of 0.012 and 0.131, respectively. Below, “confusion matrices” illustrate the proportion of assignments to the correct stimulus. The grayscale represents the proportion of the 50 responses to the call stimulus indicated on the x-axis that was estimated to be closest to the responses evoked by the call stimulus indicated on the y-axis and assigned to this stimulus. A black diagonal on a white background would indicate the assignment of all spike trains to the correct stimulus.

To assess whether auditory responses to call playbacks differed between males and females, we first performed a global analysis based on the spiking activity collected before and during call presentations. Analysis indicated that the baseline firing rate differed between males and females [GLM; 120 neurons, six types of call stimuli, five blocks, 10 presentations per block, *B*_*FR*_ vs. *S*_*FR*_; period effect: *F*_(1, 1411)_ = 200.12, *p* < 0.001; sex effect: *F*_(1, 1411)_ = 14.31, *p* < 0.001; *post-hoc* Tukey test; *p* < 0001; Figure 2A]. Presentation of call stimuli reliably evoked strong auditory responses in CLM [period effect: *F*_(1, 1411)_ = 200.12, *p* < 0.001; Figure 2A]. The Figure 3 shows representative responses to call playbacks. However, call stimuli caused neuronal activity to increase in both males and females to different extents. Whereas, the spontaneous activity rate differed between the sexes, the magnitude of call-evoked spiking activity did not (*p* = 0.32).

The previous statistical analyses used data from each single unit as a sample. However, as previously indicated, the number of recorded single units differed between individuals (range: 6–14 single units in females; 4–12 in males). Thus, to minimize the possible effects of this difference, the number of data points was reduced to match the number of individuals. We averaged all data collected within one individual. We found the same pattern of results: both a sex difference in firing rate [GLM; 14 birds, six types of call stimuli, 50 presentations, *B*_*FR*_ vs. *S*_*FR*_; sex effect: *F*_(1, 144)_ = 5.33, *p* = 0.02], a significant increase in firing rate elicited by the presentation of call stimuli [period effect: *F*_(1, 144)_ = 65.93, *p* < 0.001], a sex difference in spontaneous activity (*post-hoc* Tukey test: *p* < 0.001) with no significant difference in call-evoked firing rate (*p* = 0.61).

### Modulation of responses with stimulus repetition in males and females

To determine whether CLM neurons, as NCM neurons (Chew et al., 1995; Menardy et al., 2012, 2014), showed a decline in response magnitude with call repetition, we examined the responses of single units to repeated presentations (trials, i.e., iterations) of call stimuli. Each call stimulus was presented for five consecutive blocks of 10 consecutive trials with a 5 s-silence between two blocks.

**Figure 4:**
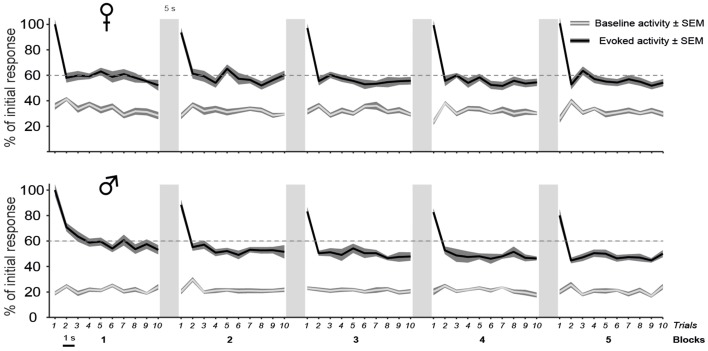
**The decline of auditory responses with call repetition is more pronounced in males than in females**. A iteration-by-iteration time course of spontaneous activity (gray lines) and responses (black lines) shown for females (top) and males (bottom). Both baseline levels and responses are expressed as a percentage of the call-evoked firing rate in Iteration 1 and are averaged over all call stimuli. The baseline firing rate remains stable over time. Call stimuli are presented as five blocks of 10 consecutive repetitions with a 1 s silence between two iterations and a 5 s silence between two blocks. Each call presentation induces an increase in activity over the spontaneous level (*p* < 0.05 in all cases). Within a block, the first call presentation evoked responses of a higher magnitude than subsequent presentations (*p* < 0.05). In males, Blocks 1 and 2 induce responses of a higher magnitude than blocks 3, 4, and 5 (*p* < 0.05 in all cases) and block 5 (*p* < 0.05), respectively.

As shown in Figure 4, call stimuli elicited a rapid response modulation that was mostly restricted to the first two presentations of each block. The response was highest during the first trial. By the 2nd repetition, it showed a ~40% decrease compared to its initial value and remained stable at this level during the following repetitions. The global analysis of spike rates collected before and during all call presentations indicated that repeated call exposure significantly affected the magnitude of the responses [trial effect: *F*_(9, 12699)_ = 122.4, *p* < 0.001; interaction between period and trial factors: *F*_(9, 12699)_ = 122.4, *p* < 0.001] with no change in the spontaneous activity rates over time (*post-hoc* tests, *p* > 0.05) and no difference between males and females [no significant interaction between trial and sex factors: *F*_(9, 12699)_ = 1.2, *p* = 0.29]. All call presentations evoked significant changes in the firing rate (period factor; *post-hoc* tests, all *p* < 0.001) with significant differences in response magnitude between the first trial and the successive ones (*post-hoc* tests comparing trial 1 to the trials 2–10, all *p* < 0.001).

Although the delay between two blocks of 10 trials or before the presentation of a new call stimulus had the effect of resetting the firing rate to the higher initial level in both sexes, the repetition of blocks however affected response magnitude differently between males and females [block effect: *F*_(4, 5644)_ = 37.8, *p* < 0.001; significant interaction between block and sex factors: *F*_(4, 5644)_ = 8.3, *p* < 0.001; Figure 4]. The pattern of habituation was similar across the five blocks in females (between blocks comparisons; *post-hoc* tests; all *p* > 0.05). In contrast, the mean firing rate showed a gradual decrease with block repetition in males. Block 1 drove significantly higher responses than blocks 3, 4, and 5 (between blocks comparisons; *post-hoc* tests: all *p* < 0.001).

Additionally, the analysis allowed us to examine whether the time course of changes in firing rate reflected the influence of both the sex of the subject and the type of call stimulus. In both sexes, similar modulations were induced by the repetition of the different types of call stimuli [interaction between period, block number, stimulus type and sex factors: *F*_(20, 5644)_ = 1.08, *p* = 0.37].

Therefore, the main result of the analysis of the dynamic changes in response magnitude during call repetitions is a more pronounced decline of auditory responses in males than in females.

### Strength of responses to the variety of call stimuli in males and females

To characterize call processing within the CLM, we evaluated the strength of responses driven by the six call types by calculating an index, *Ri*, that normalized the data with the sum of the spontaneous rate and the call-evoked rate. As expected on the basis of spike rate data, call stimuli drove CLM neurons more vigorously in males than in females [GLM; 120 neurons, six types of call stimuli; *F*_(1, 115)_ = 20.33, *p* < 0.001; Figure 2B]. All call types evoked higher responses in males than in females (Tukey-Kramer test, *p* < 0.01 in all cases). Therefore, the degree of excitation provided by call stimuli differed between males and females. Hence, whereas the level of spontaneous activity was higher in females than in males, call-evoked responses were stronger in males than in females.

**Figure 5:**
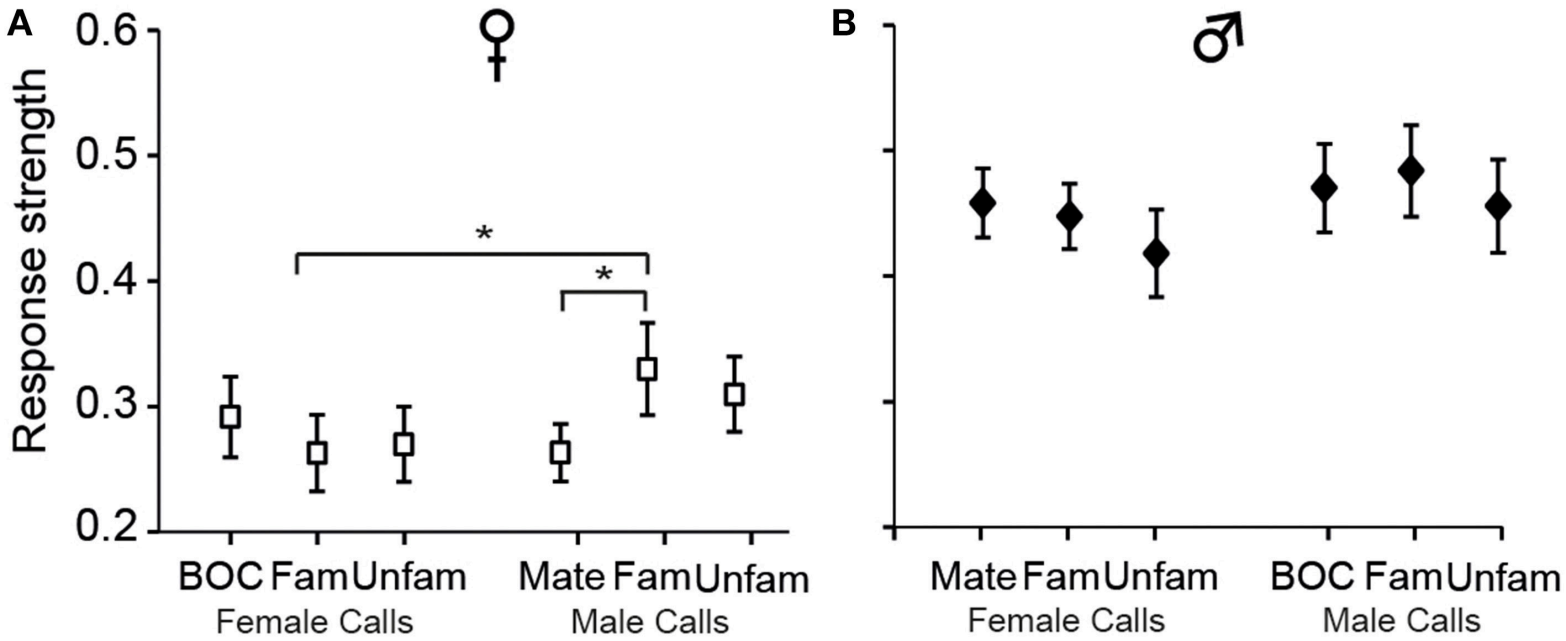
**The strength of responses varies among call stimuli in females (A) but not in males (B)**. Each bar represents the mean ± SEM. (^*^p < 0.05).

Analyses of *Ri* values also indicated that call stimuli differentially drove CLM neurons [*F*_(5, 575)_= 5.12, *p* < 0.001; when one averaged data point per bird and per call stimulus: *F*_(5, 60)_ = 2.58, *p* = 0.03; Figure 5]. *Post-hoc* tests indicated that, in females, the calls of familiar males evoked greater response strength than the mate's call (*p* < 0.03) or the calls of familiar females (*p* < 0.03). No such differences were observed in males (all *p* > 0.05). These results therefore provide evidence that variations in response strength across call types differ between males and females. Only in females, response strength showed differences between certain call stimuli, those of “familiar individuals.”

### Variation in call-evoked responses among CLM single units in males and females

Analyses of responses to various auditory stimuli carried out above were based on population data, leaving variations in responses across individual single units unclear. In both sexes, most neurons responded to a broad range of call stimuli. We computed the number of neurons that were responsive to the call stimuli. A neuron was considered responsive to a given call stimulus when it met both the following arbitrary criteria: the *Ri* value was >0.15 and the average spiking rate exceeded the average baseline level by 2 SD during at least one 10 ms bin. However, the number of responsive neurons was more pronounced in males than in females. On average, a call stimulus elicited auditory responses in a larger subset of neurons in males than in females (out of 60 single units in both sexes: 51.7 ± 1.6 vs. 42.0 ± 2.5; χ^2^ = 3.04; *p* = 0.012; range: 47–54 cells vs. 37–47). Neurons responded to 5.17 ± 0.18 call types (out of the six call stimuli; data were averaged per type of call stimuli) in males and to 4.25 ± 0.24 in females (χ^2^ = 3.05, *p* = 0.003). We noticed that about half of the cells responded significantly to all types of call stimuli (34/60 in males vs. 24/60; χ^2^ = 3.34; *p* = 0.07).

To examine whether the reduced number of call stimuli driven responses in females compared to males made neurons in females more discriminative than neurons in males, we computed a nonparametric measure, the AF index, used in recent studies to compare the distribution of response magnitude to song stimuli between various telencephalic auditory areas, including the CLM (Jeanne et al., 2011; Meliza and Margoliash, 2012). In the present study, the index takes into account responses to the six call types. As shown in Figure 2D, the range of index values was similar in both sexes. Also, most neurons in both sexes exhibited low index values indicating that neurons responded with different spike rates to each of the six stimulus categories, but with a low degree of variations across stimuli. However, many more neurons in females exhibited a very low value. On average, neurons in females had a lower AF value than neurons in males (0.06 ± 0.01 vs. 0.102 ± 0.01; *t-*test, *t*_118_ = 1.98, *p* = 0.04) indicating that spike rate values of CLM neurons are more similar between call stimuli in females than in males.

Our results therefore suggest that neurons in the CLM of males, in comparison of neurons of females, responded to a broader range of call stimuli and were more discriminative.

### Temporal characteristics of call-evoked discharges in males and females

**Figure 6:**
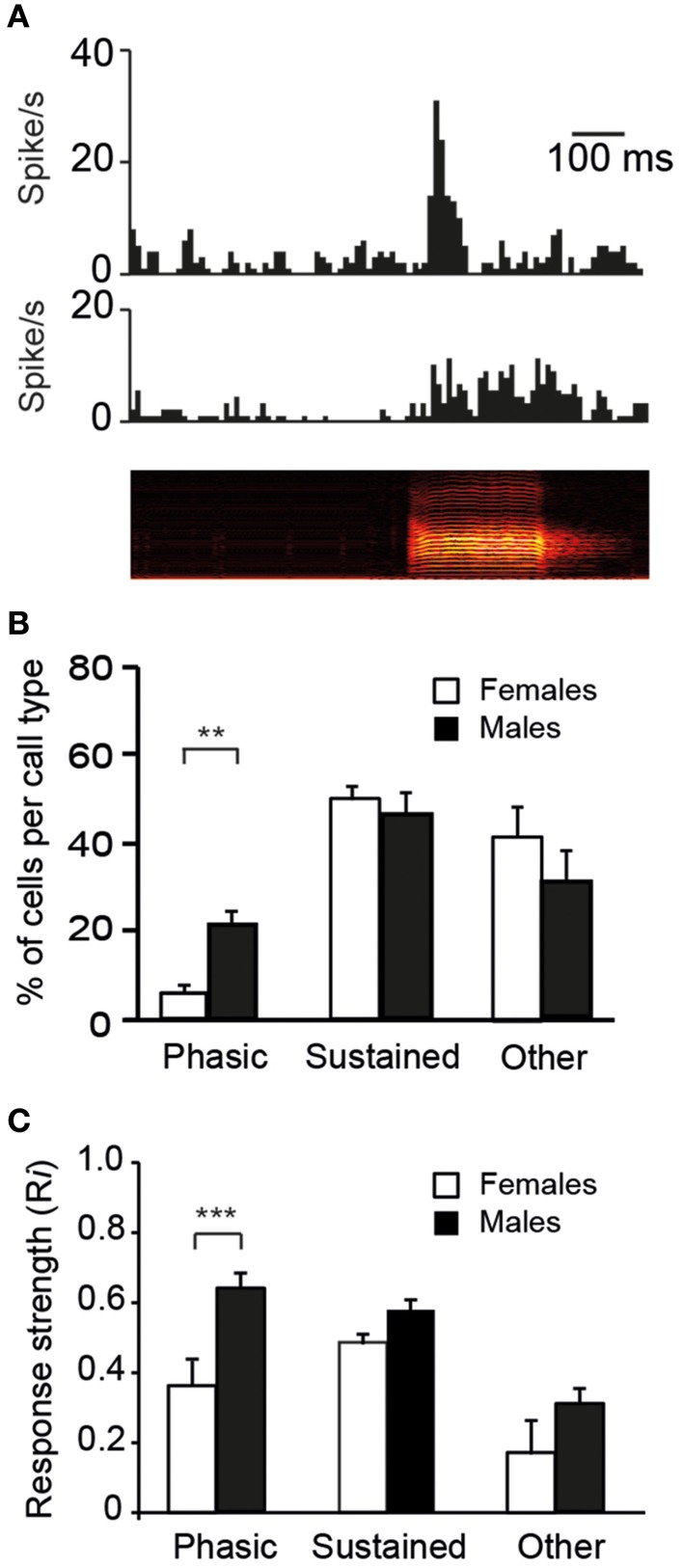
**The diversity in responses to calls by single units in the CLM**. **(A)** Representative examples of a phasic (top) and a sustained (middle) response to the playback of a call stimulus (bottom). The call stimulus is depicted as a spectrogram. **(B)** The mean proportion of cells per call type within each of the three categories of responses: phasic, sustained, and other which includes an absence of response and/or an unidentified profile of response (^**^p < 0.01). In response to each call type, we quantified the number of cells according to the profile of the response. **(C)** Average response strength. Call stimuli induce stronger phasic responses (*Ri* values) in males than in females (^***^p < 0.001). Each bar represents the mean ± SEM.

Auditory responses may also differ between males and females with respect to their temporal characteristics. On the basis of visual inspections of PSTHs, two response profiles were mainly observed: a striking increase in activity at stimulus onset that was followed by a rapid decline in firing rate or a sustained activation over the entire duration of the call stimulus (Figure 6A). For each of the 10 call stimuli, we thus quantified the number of cells exhibiting phasic or sustained responses and the number of cells that did not satisfy the criteria, i.e., that were non-responsive to any call stimuli and/or that showed neither phasic nor sustained responses (three categories of responses). Nearly all cells (55/60 cells in males and 51/60 cells in females) responded to at least one call stimulus by either phasic or sustained excitation (the 14 remaining cells were categorized as “other”). We also quantified the proportion of cells displaying phasic, sustained, or other responses in response to the call stimuli (Figure 6B). The average proportions of cells in the three categories differed between the sexes (χ^2^ = 7.3; df = 2, *p* = 0.02). A lower proportion of CLM cells exhibited a phasic increase in response to the call stimuli in females than in males (χ^2^ = 12.1; df = 1, *p* = 0.005). On average, 2.4 and 12.4 cells per call stimulus showed a phasic response in females and males, respectively (range: 0–4 in females; 10–16 in males). Moreover, a sex difference was also observed in the strength of phasic responses: *Ri* values were significantly lower in females than in males (total number of phasic responses: 24 in females vs. 124 in males; *t*_147_ = 4.3; *p* < 0.001; Figure 6C). Therefore, phasic responses were less frequently observed and less strong in females than in males. It can be noticed that the averaged *Ri* value (calculated over the entire duration of the call stimulus) of phasic responses was similar to that of sustained responses, although phasic responses were shorter in duration (Figure 6C). This indicates that, unlike the vast majority of neurons in females, a number of neurons in males were able to exhibit a vigorous increase in activity in response to call playback. Overall, 61% of the neurons recorded in males showed such strong phasic responses to at least one call stimulus.

**Figure 7:**
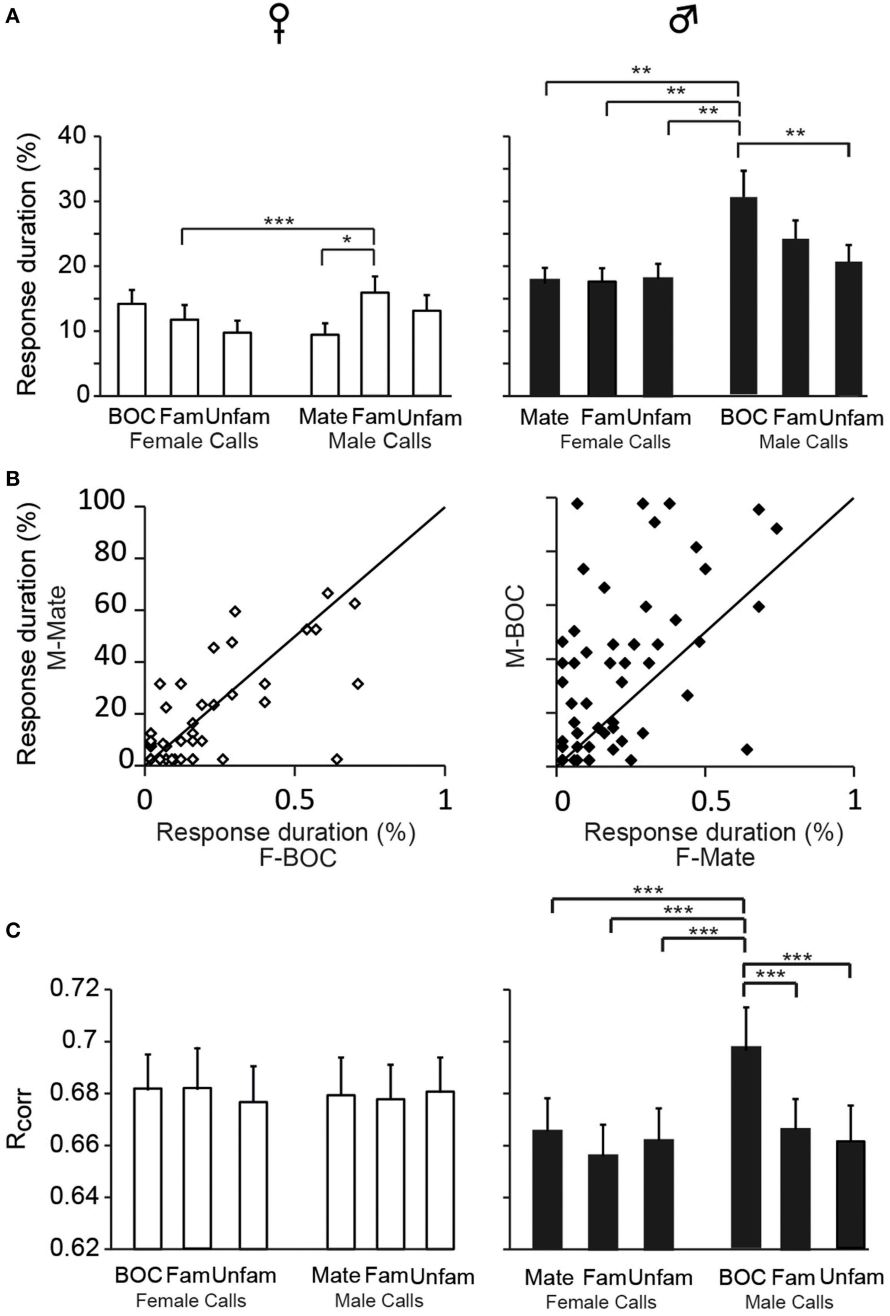
**Sex differences in the temporal features of call-evoked responses**. **(A)** Duration of responses to the variety of call stimuli in females (left) and in males (right). Based on PSTHs built from spike trains, the number of 10 ms bins in which activity exceeds the baseline level by 2 SD is quantified and expressed as a percentage of the total duration of the call presented. In males, the bird's own call, which is the mate's call in females, induces longer responses than most other call stimuli (^*^p < 0.05; ^**^p < 0.01). **(B)** Within-cell comparisons of the duration of responses to the calls of the two partners: the mate's call and the bird's own call. The same set of stimuli was presented to both partners. Each dot represents a single cell. The diagonal line represents equal responsiveness to both stimuli. Points to the left of the diagonal line are biased toward the male's call while points to the right are biased toward the female's call. In males, a majority of cells show longer responses to the male's call than to the female's call. **(C)** The computed correlation index between spike trains (*R*_*corr*_) elicited by repetitions of the same stimulus does not differ between call stimuli in females. In males, the bird's own call yields a higher average *R*_*corr*_ value than all other call stimuli (^***^p < 0.001 in all cases). Each bar represents the mean ± SEM.

The assignment of responses to categories, phasic or sustained, was based on criteria that we defined on the basis of visual inspections of PSTHs. We also evaluated the duration over which spiking activity was significantly increased relative to the call duration. To this end, we quantified the cumulative number of 10 ms time bins in which activity exceeded the baseline level by 2 SD and then, we calculated the proportion of 10 ms bins relative to the total number of 10 ms time bins of the call. This quantification allows evaluating the degree of call-evoked excitation provided by call stimuli in the temporal domain but does not contribute to reveal any temporal structure in responses. Overall, on the basis of the proportion of 10 ms bins during which the activity was significantly increased, responses to call stimuli were shorter in duration in females than in males [GLM; 120 neurons, six call stimuli; *F*_(1, 118)_ = 125.17, *p* < 0.001; when one averaged data point per bird: *F*_(1, 12)_ = 30.84; *p* < 0.001; Figure 2C] and differed between call types [*F*_(5, 590)_ = 10.17, *p* < 0.001; when one averaged data point per bird: *F*_(5, 60)_ = 4.11; *p* = 0.002; Figure 7A]. In accordance with the previous results, assessing temporal characteristics of responses revealed a lower degree of excitation of neurons by call stimuli in females than in males. Results also indicated that the bird's sex had an influence on how response duration varied with call types [interaction between sex and call type factors: *F*_(5, 590)_ = 6.00, *p* < 0.001; when one averaged data point per bird: *F*_(5, 60)_ = 2.76; *p* = 0.02]. *Post-hoc* tests indicated that, in females, the call of familiar males elicited longer changes in activity than the mate's call (*p* = 0.02) or the call of familiar females (*p* < 0.001). In males, responses to playback of the bird's own call were longer than responses to all other calls (all *p* < 0.002) except the call of familiar males (*p* = 0.12). As shown in Figure 7B, when the duration of responses to the mate's call was plotted against that of responses to the bird's own call in females and males, the range of values for the bird's own call was clearly broader in males. Therefore, beside longer responses in males than in females, how response duration varied across call stimuli differed between males and females.

We also used a common approach that did not depend on identifying differences in features either between stimuli or between responses, by calculating the amount of MI between stimuli and spike trains (Hsu et al., 2004; Huetz et al., 2006; Schneider and Woolley, 2010; Tremere and Pinaud, 2011; Menardy et al., 2012, 2014). MI captures differences between the spike trains elicited by different stimuli by evaluating how the response patterns are both reproducibly similar for repeated presentations of the same stimulus and reproducibly different between presentations of different stimuli. Female and male calls differed in their acoustic structure, especially in their duration. To evaluate the amount of information conveyed by the spike trains of each neuron, MI values for male and female calls were calculated separately. Comparisons revealed that mean MI values were significantly lower in females than in males [RM ANOVA; 120 neurons; two types of call stimuli, male vs. female calls; female calls: *F*_(1, 118)_ = 6.71, *p* = 0.01; when MI values were averaged over units per subject; *F*_(1, 12)_ = 4.96, *p* = 0.04; male calls: *F*_(1, 118)_ = 13.04, *p* < 0.001; when MI values were averaged over units per subject; *F*_(1, 12)_ = 6.29, *p* = 0.03; Figure 2E], but did not differ between male and female calls in either sex (all *p* > 0.8). The temporal structure of spike trains in females thus transmitted a lower amount of information than those of neurons in males. Female neurons might have a low MI value because spike trains lack distinctive call-specific patterns or reproducible patterns in response to repeated presentations of the same stimulus. To address this issue, we quantified the spike-timing reliability of responses to a given call stimulus using the *R*_*corr*_ index (Schreiber et al., 2003; Huetz et al., 2006). Based on mean *R*_*corr*_ values (Figure 2F), neurons in both males and females displayed trial-to-trial reliable responses. Results indicated that *R*_*corr*_ values differed between sexes [GLM; 120 neurons, six call stimuli; sex factor: *F*_(1, 118)_ = 466.7, *p* < 0.001], with the mean *R*_*corr*_ value being higher in females than in males (Figure 2F). *R*_*corr*_ values also differed between call types [*F*_(5, 590)_ = 4.39, *p* < 0.001], with a significant interaction between call types and the bird's sex [*F*_(5, 590)_ = 3.57, *p* = 0.003; Figure 7C]. *Post-hoc* analyses did not reveal any difference in mean *R*_*corr*_ values between call stimuli in females. In contrast, in males, the bird's own call led to a higher mean *R*_*corr*_ value than all other call stimuli (*p* < 0.001 in all cases). Therefore, as data indicated higher overall *R*_*corr*_ values with lower overall MI values in females than in males, they suggest that responses in females were more consistent but showed a lower degree of discrimination across call stimuli.

In males, analysis of *R*_*corr*_ values indicated that responses evoked by the bird's own call showed the highest degree of robustness suggesting that the encoding of this call by CLM neurons differed from that of other call stimuli. To address this issue, we analyzed the proportion of assignments to the correct call stimulus obtained in the confusion matrix when investigating the MI between neural responses and male calls. The analysis revealed that the proportion of correct assignment differed among male call stimuli [RM ANOVA; 60 neurons, five call stimuli including the two familiar and unfamiliar calls; *F*_(4, 236)_ = 7.66, *p* < 0.001; bird's own call: 50 ± 3%; familiar calls: 38 ± 2 and 33 ± 2%; unfamiliar calls: 46 ± 2 and 36 ± 2%]. Spike trains evoked by the bird's own call were more often classified as belonging to this call stimulus than spike trains evoked by the other call stimuli (*post-hoc* tests: all *p* < 0.008 except one unfamiliar call (*p* = 0.76). Therefore, results based on the analyses of temporal structure of spike trains suggest that the neural responses of the CLM in males tend to better represent the bird's own call than other male calls. We also examined whether the proportion of correct assignment differed among male call stimuli in females. The analysis did not reveal any difference [*F*_(4, 236)_ = 1.48, *p* = 0.2; mate's call: 38 ± 2 and 33 ± 2%; unfamiliar calls: 37 ± 2 and 39 ± 2%]. Therefore, in females, the mate's call (the bird's own call in males) was not differentially encoded than the other male calls. In females, the proportion of correct assignments did not differ between female calls that included the bird's own call [*F*_(4, 236)_ = 0.59, *p* = 0.67, bird's own call: 36 ± 2%; familiar calls: 38 ± 2 and 38 ± 2%; unfamiliar calls: 39 ± 2 and 34 ± 2%].

## Discussion

Using the same set of male and female distance call stimuli to characterize the auditory responsiveness of CLM neurons allowed us to highlight multiple sex differences in the encoding properties of CLM neurons. While neurons in both males and females responded to a broad range of call stimuli, how auditory responses varied across call types differed between males and females, with a special sensitivity to the bird's own call in males. Auditory processing of distance calls in the CLM therefore exhibited sex-dependent variations.

### Sex differences in the response properties of auditory neurons in the CLM

We investigated sex differences in an auditory region of a species known to show a high degree of sexual dimorphism in singing behavior. Numerous studies have assessed sex differences in this species at various anatomical levels of the song control pathways, in order to examine, in particular, to what extent morphological organization fits the structure-function rule. In contrast, it is only recently that the possibility that sex differences in zebra finches could extend to auditory regions has received attention (Pinaud et al., 2006; Yoder et al., 2015). Here, we contribute to the evidence that sexual dimorphism also occurs in higher-order processing areas within the auditory system.

Call-evoked responses showed a broad range of differences between males and females. At first, response strength was found to be lower in females than in males, regardless of the information encoded by the call stimulus. Such a difference has been recently observed in another brain region, the NCM, while presenting conspecific or heterospecific songs (Yoder et al., 2015), suggesting that sex differences in auditory responsiveness are not limited to a particular pallial auditory area or a single type of vocalization. Importantly, the lower capacity of neurons to be driven by call stimuli in females relative to males was not due to a lower call-evoked firing rate, but, on the contrary, to higher spontaneous activity, suggesting that the sex difference in auditory responsiveness could result from a difference in the physiological properties of CLM neurons. Further work will be required to examine to what extent the intrinsic properties of neurons in the CLM, as well as in other auditory areas such as the NCM, depend on the bird's sex. Another property of CLM neurons was found to be affected by the bird's sex: the dynamic range of responses during the repetition of calls. In the CLM, response magnitude mostly declined between the first and second occurrence of the stimulus and then remained fairly stable. In females, a few seconds of silence reset firing to initial levels, suggesting that only short-term plastic changes in response magnitude took place with call repetition. In males, a more prolonged decrease was observed: although firing activity was reset, the pattern of response modulation showed a fairly slight decrease with repetition. Consequently, by the 15th presentation, females exhibited a shallower adaptation rate than males. Such a sex difference in adaptation rate has also been described in the NCM of awake zebra finches (Yoder et al., 2015). The neural mechanisms responsible for these plastic changes in responsiveness, which remain largely to be defined, might therefore depend on the bird's sex. In the present study, the repeated presentation of only one example of each individual's call stimulus at a regular rate therefore led us to reveal sex differences in dynamic changes of responses. However, one could wonder to what extent the other sex differences in response properties reported here would have been observed if various examples of each individual's call had been randomly and irregularly presented. Although it remains to be investigated, we could suspect that neither rapid nor more prolonged changes in response magnitude would have occurred with call presentations leading neurons to exhibit greater responses, especially in males. Consequently, one may assume that at least, an even greater sex difference in response strength and, speculatively, in response duration would have been observed.

Other response features highlighted sex differences in call-evoked responses. Neurons in males responded to more call types and presented more distinctive spike rates across call types than neurons in females since AF values were higher in males than in females. With regards to the profile of responses, phasic or sustained, fewer neurons in females compared to males were found to respond with a phasic profile to distance calls. Also, the degree of reliability between spike trains, based on the computed correlation index was, on average, higher in females than in males and did not vary across call stimuli in females. Moreover, the amount of information conveyed by spike trains, as indicated by MI values, was lower in females than in males. Therefore, the present study provides several evidence that males and females differ in how call stimuli are encoded in the CLM.

### Sex differences in the auditory processing of call stimuli by the CLM

Distance calls contain enough information to allow individuals to recognize their mate or familiar individuals (Zann, 1984; Vignal et al., 2004; Forstmeier et al., 2009; Vignal and Mathevon, 2011). Secondary regions of the songbird auditory telencephalon are considered as playing a critical role in the process of discrimination of vocal communication sounds and as being important sites for the storage of information about the bird's auditory experience (Gobes et al., 2010; Thompson and Gentner, 2010; Jeanne et al., 2011; Bolhuis and Moorman, 2015). The neuronal correlates of call-based discrimination have already been found in another secondary auditory region, the NCM, in zebra finches (Woolley and Doupe, 2008; Gobes et al., 2009; Menardy et al., 2012). The proportion of neurons that encode the various categories of vocal communication sounds, including calls, varies among secondary auditory regions (Jeanne et al., 2011; Elie and Theunissen, 2015). Also, the sensitivity of neurons to familiar and unfamiliar vocalizations differs between secondary auditory regions (Meliza and Margoliash, 2012). In the present study, we did not provide any evidence that the responses of CLM neurons distinguish between the calls of unfamiliar individuals and those of familiar ones, including the mate's call. However, this issue will require further investigation. Given that our study was performed under anesthesia, one cannot exclude the possibility that discrimination among call types based on the degree of familiarity of the call occur in the CLM of awake birds.

Nevertheless, this report suggests that, in females, the CLM could contribute discriminating among distance calls, especially among calls of familiar individuals. The analysis of responses based on spike rate, i.e., response strength and response duration, revealed differences between the calls of familiar males and those of familiar females or the mate's call. Thus, experiencing the calls of familiar individuals, including the mate and males and females of other pairs, might have specifically affected how these calls were represented in the CLM. Further work will be required to support this assumption. It remains unclear whether passive exposure or social interactions are required for this discrimination. Additionally, the AF measure indicates that none of the neurons in females were selective for a given call type, responding to many call types. This suggests that the information about the caller identity could be encoded at the population level.

In males, responses in the CLM to call stimuli did not reflect, at the neuronal level, a behavioral preference for the mate's call or for calls of familiar females over unfamiliar ones. It should be mentioned that males are able to behaviorally discriminate among female calls only if they are in the presence of an audience (Vignal et al., 2004). Therefore, one cannot exclude the possibility that neuronal activity in the CLM is influenced by social conditions, as has been observed in the NCM of awake paired males (Menardy et al., 2014).

In the present study, our results in males pointed rather to a higher sensitivity to the bird's own call according to temporal features of responses. This call elicited more long-lasting changes in activity in proportion of the call duration than most of the other call stimuli. Spike timing was the most reliable when this call was presented. Also, the temporal structure of spike trains, assessed by the percentage of assignment to the correct stimulus when the amount of MI was quantified, distinguished the bird's own call from the other call types. Beside these temporal features, analyses of the spiking rate did not reveal any difference between the bird's own call and other calls. Thus, the bird's own call could be encoded by temporal information rather than firing rate. In contrast, in females, the same stimulus, i.e., the mate's call, did not differ from the other call types by its degree of reliability or the percentage of correct assignment, the duration of responses tended to be the shortest. Importantly, in females, responses driven by the bird's own call did not differ from responses to other call stimuli in any respect.

### Hypotheses on CLM function in male and female zebra finches

Previous research on immediate early gene induction in the avian ascending auditory pathway has revealed sex differences in the processing of calls (Avey et al., 2008; Gobes et al., 2009). Here, the recording of single-unit responses in paired male and female zebra finches during the presentation of distance calls provides additional support for the hypothesis that the processing of vocal communication sounds in the auditory pallium is sexually dimorphic. This implies that the CLM could serve different functions in males and females. Hypothetically, the great degree of sexual dimorphism in both auditory vocal behavior and experience, which are intimately related in zebra finches, could be associated with a sex difference in CLM function. Adult females do not sing and their distance calls are not learned (Zann, 1985; Forstmeier et al., 2009), but they are able to identify individuals, such as their mate, on the basis of their vocalizations (songs or calls). In females, the CLM could be an auditory region contributing to the perception of natural vocalizations and the storage of information about the bird's auditory experience. Differences in response strength between familiar male and female calls provide support for this assumption. In males, the CLM could contribute to the auditory processing of self-generated vocalizations. In addition to the present study, which shows the distinctive encoding of the bird's own call, other studies have led to the assumption that the CLM could transmit information about singing-related auditory feedback to the song system. Neurons in the CLM of awake male zebra finches show a change in their activity in response to song playback and during singing (Bauer et al., 2008). A subset of CLM neurons in males exhibit a preference for the bird's own song over other conspecific songs (Bauer et al., 2008). Also, some neurons are highly feedback-sensitive, in that they respond vigorously to song perturbations, but not to unperturbed songs or perturbed playbacks (Keller and Hahnloser, 2009). In males, both song and distance calls are learned vocalizations (Zann, 1984; Forstmeier et al., 2009). Indeed, the adult form of the distance call results from a tutor-guided learning process and its learned features are controlled by nuclei that control song production (Simpson and Vicario, 1990). In the absence of a tutor, the distance call develops but exhibits female-like features. Thus, the male-specific sensitivity to the bird's own call and, beyond that, sex differences in the auditory response properties of CLM neurons, might be related to the greater degree of sexual dimorphism in vocal behavior and related processes, including the learning-dependent sensorimotor integration that exists in zebra finches. Obviously, other factors could contribute to the sexual dimorphism in the auditory processing of distance calls in zebra finches, including hormonal state (Maney and Pinaud, 2011).

To summarize, in agreement with a growing number of studies, the present work provides support for the assumption that, in zebra finches, auditory processing of distance calls in the CLM exhibit sex differences suggesting a sexual dimorphism in the function of the CLM. In songbirds, the well-known sexual dimorphism in vocal production could therefore be extended to the auditory processing of vocal communication sounds.

### Conflict of interest statement

The authors declare that the research was conducted in the absence of any commercial or financial relationships that could be construed as a potential conflict of interest.
